# Mixed-methods service evaluation of a multidisciplinary inpatient programme for functional neurological disorder and non-epileptic attack disorder

**DOI:** 10.1192/bjo.2021.106

**Published:** 2021-06-18

**Authors:** Peter Denno, Samir Sholapurkar, Elizabeth Mallam, Dane Rayment

**Affiliations:** 1 North Bristol NHS Trust; 2 North Bristol NHS

## Abstract

**Aims:**

To evaluate a multidisciplinary inpatient treatment programme for Functional Neurological Disorder (FND) and Non-Epileptic Attack Disorder (NEAD), focussing on clinical effectiveness and patient experience. To produce recommendations for service development and future evaluation.

**Method:**

We conducted a service evaluation of the multidisciplinary inpatient programme for FND and NEAD at the Rosa Burden Centre. We contacted all inpatients discharged between December 2019 and March 2020 via telephone in August/September 2020. Quantitative outcomes were gathered on quality of life and psychological distress using the EQ-5D-5L and Core10 tools. Scores were compared to those gathered routinely at admission and discharge, using Wilcoxon's test for differences. Qualitative feedback on patient experience was gathered using open-ended prompts, and thematic analysis of this data was conducted independently by two researchers. Approval was gained from Southmead Clinical Audit Department (CE10237).

**Result:**

19 of 22 patients successfully completed the service evaluation. Quantitative results tended toward improvement on all measures between admission and discharge. Following discharge, there was a mixed pattern - sustained improvement in overall quality of life, but regression in other scores. Improvement in overall quality of life between admission and follow-up was statistically significant (p = 0.012, Z = 2.52). Changes in psychological distress (Core10) were also statistically significant, reducing between admission and discharge (p = 0.004, Z= –2.84) and increasing between discharge and follow-up (p = 0.016, Z = 2.42). Changes in other scores were not statistically significant at the p < 0.05 level. Qualitative results highlighted the value of the individual therapies offered, the multidisciplinary approach, and the supportive environment. Participants reported improved understanding of their diagnosis, and of self-management strategies. There was demand for greater access to psychological therapies, and increased provision of follow-up post-discharge. Some expressed dissatisfaction with the ward round format and excess “down-time”. The programme was described as a “turning point” for 9 participants.

**Conclusion:**

Quantitative results suggest the programme is associated with global improvement in quality of life, and post-discharge, some benefits are sustained while others are transient. However, interpretation is limited by sample size. We recommend further evaluation with a larger sample to replicate findings, assess effect sizes, and assess which patients or symptoms benefit most. To support this, we recommend improved collection of outcome measures, including routine collection of follow-up data. Positive qualitative findings highlight the strengths of the service and its value to patients. Recommendations for service development include recruiting a psychologist to provide further psychological therapy sessions; expanding the nurse-led follow-up service; and adjustments to the ward round format and activity programme.

